# Evidence from an Applied Health Research Question (AHRQ): Health care utilization of publicly funded rehab services for patients post COVID-19 diagnosis.

**DOI:** 10.23889/ijpds.v7i3.2085

**Published:** 2022-08-25

**Authors:** Kim Mcgrail, Fiona Clement, Michael Law

**Affiliations:** 1 The University of British Columbia; 2 University of Calgary

## Objectives

Population data scientists are committed to research that has public value. Much of this research is applied; it is undertaken in partnership with the public, patients, families, as well as policy- and decision-makers. Working directly with policy-makers (who are often also data providers) has advantages, but presents challenges as well.

## Approach

We offer four provocations to stimulate thinking about the relationship between research and the “systems” that research is trying to influence. These provocations include: 1) assessing the implications of “partnership” and who is expected to change or accommodate others’ views, and how this affects researchers’ ability to challenge current practice; 2) challenging the emphasis on short-term over longer-term challenges in systems; 3) moving beyond post-implementation evaluations of policies; and 4) critiquing the current project-specific orientation to assessing return on investment (ROI).

## Results

The current focus on partnership in applied research tends to suggest that it is researchers who need to be empathetic to the timelines and needs of policy makers. True relationships, however, are bi-directional, and more importantly need to be open to tough conversations and constructive feedback. Further, focusing on priorities of “systems” will emphasize short-term issues. These are important to address, but can crowd out more systemic and structural considerations. This leads to researchers often engaged in post-implementation evaluation where they have had little involvement in policy or intervention design, which may not be evidence-based. Finally, a focus on single-project ROI will tend to undervalue riskier – but also potentially more rewarding – research.

## Conclusion

It is important to recognize that valuable research might challenge current thinking and practice, and/or address issues that are not short-term priorities. More early testing of policies before broad implementation will advance evidence. ROI should be viewed as an emergent property rather than an attribute of each individual project.

